# Antiobesity Potential of Selected Latin American Edible Plants: A Review

**DOI:** 10.1007/s11130-026-01467-3

**Published:** 2026-02-10

**Authors:** Talía Hernández-Pérez, Octavio Paredes-López

**Affiliations:** Departamento de Biotecnología y Bioquímica, Centro de Investigación y de Estudios Avanzados del Instituto Politécnico Nacional, Irapuato, Gto México

**Keywords:** Obesity, Satiety, Overweight, Plant foods

## Abstract

The antiobesity potential of some plants refers to their capacity to suppress appetite, inhibit digestive enzymes and interfere in fat absorption to control or reduce weight and preferentially without side effects. Healthy dietary habits, in addition to exercise, improve or delay the complications associated with obesity (*e.g*., prevention and control of type 2 diabetes, hypertension, fatty liver disease, and obstructive sleep apnea), and consequently quality of life. An effective strategy to maintain a healthy body weight is including food plants in the daily diet. Many food crops comprise bioactive compounds with a wide range of health benefits, such as unsaturated fatty acids, soluble and insoluble fiber, pigments (chlorophyll, betalains, carotenes, anthocyanins), phenolic compounds, flavonoids, and stilbenes, among others. Due to the side effects of synthetic antioxidants and antiobesity drugs, scientists are now focusing on natural products which produce better effects and less side effects. Nowadays, research is aiming on the outstanding characteristics of crops that have been used from ancient times in different regions worldwide, including Latin America. In this review, we revised the state of knowledge of the nutraceutical and antiobesity properties of different native cultivars of Latin America, including, quinoa (*Chenopodium quinoa*), chia (*Salvia hispanica*), amaranth (*Amaranthus hypochondriacus*), nopal (*Opuntia* spp.), avocado (*Persea americana*), pineapple (*Ananas comosus*) and cacao (*Theobroma cacao*). We hope that the selected food sources from this region will have a better use in the control of overweight and obesity.

## Introduction

Obesity is one of the major pandemics worldwide and nowadays is the leading cause of several chronic degenerative diseases. It is characterized by a body mass index (BMI) above 30 kg/m^2^ in adults [1]. A balanced diet and a healthy lifestyle are safe and effective strategies to prevent the onset of obesity. Since 1990, obesity in adults and adolescents has doubled and quadrupled, respectively. In 2024, more than 1 billion people presented obesity, and 1 of 8 deaths attributed to non-communicable diseases are driven by overweight or obesity, mostly due to diabetes, stroke, coronary heart disease, and cancer. In addition, the increasing incidence of this condition in young people is also alarming. It is expected that for 2035 more than 1.77 billion people will present overweight, and 1.53 will have obesity, which represents in total 54% of all adults around the world. This percentage is considered a problem in high-income countries, although nowadays is also rising in low- and middle-income countries as well [2]. Some of the traditional treatments of obesity include low-carbohydrate diets, insulin-sensitizing drugs, and physical exercise.

On the other hand, there are key studies describing very clearly the effects after the supplementation of daily diets with edible foods native to Latin America; such positive effects on obesity and its comorbidities have been shown by lipid and glycemic control variables, among other parameters [3, 4]. The regular intake of native food crops from Latin America may help in the prevention or treatment of non-communicable diseases, such as cancer, type 2 diabetes, hypertension, and obesity [5, 6].

Quinoa, chia and amaranth are some of the rediscovered Latin-American crops with outstanding agronomical and nutraceutical properties. These last properties are due to components with diverse functions like antioxidants, anti-inflammatory, anti-hypertensive, anticancer and antiobesity capacity. Also, other crops from this region have similar potential like nopal (*Opuntia* spp.), avocado, pineapple and cacao, among other important regional cultivars [7]. They are rich sources of fatty acids, sterols, phenolic compounds and dietary fiber which can increase satiety. In addition, they offer outstanding features for the food and nutraceutical industries due to their functional components [8].

The regular intake of traditional food plants from Latin America has been increasingly associated to the prevention or mitigation of chronic diseases, like type 2 diabetes, cancer, hypertension, dyslipidemia, inflammation, among others [9]. Thus, this review is focused on the scientific in silico, in vitro and in vivo novel evidence of the different potential benefits of some selected food sources from this region on the treatment and control of overweight and obesity.

## Latin American Food Crops with Antiobesity Capacity

### Quinoa

**Fig. 1:**
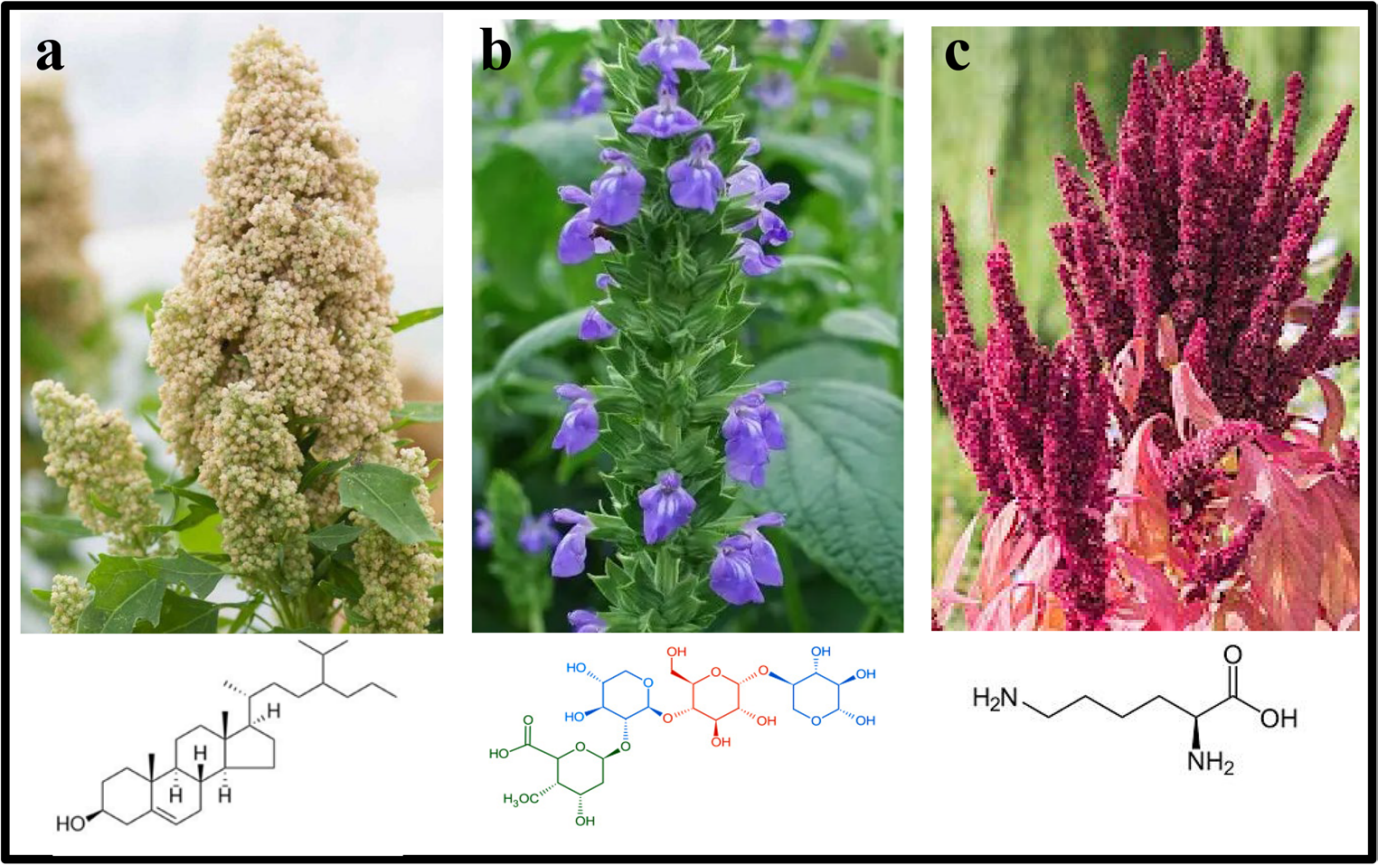
Quinoa phytosteroids (**a**), chia mucilage (**b**) and amaranth leucin (**c**) with antiobesity potential

Quinoa (*Chenopodium quinoa*) is a pseudocereal originated in the Andean region of South America thousand years ago (Fig. 1a). It is available in a range of colors like yellow, white, red and purple. Its pigmentation comes from its phytochemical composition; levels of bioactive compounds, mainly flavonoids, phenolics and betalains, which play a key role not only in the different seed colors, but also in its antioxidant potential. A more than outstanding characteristic of this plant, based on its agronomic performance among other facts, has given a recognition as the crop of the XX1st century with the distinguished potential to be among the 10 crops to play a key role for feeding the worldwide population [10]. This seed has a valuable nutraceutical message due to its superior balance of carbohydrates, lipids, proteins, vitamins, saponins, minerals, phenolics, glycine betaine, phytosterols, and betalains [11]. Quinoa is also an excellent source of dietary fiber, which contributes to its functional properties including management of weight levels in humans mostly from South America; they incorporate quinoa in their frequent diets [12].

Quinoa proteins contain the nine essential amino acids, and is a gluten-free grain, that have exerted in vitro and in vivo medicinal properties to treat certain chronic degenerative diseases, *e.g*., obesity, diabetes, cardiovascular diseases, and some types of cancer [13]. It is worth to mention that saponins are present in the outer layers of the seed and because of their composition usually show a bitter taste with a known antinutritional role and especially with a minor inhibition of weight increase [14].

In mice, quinoa polyphenols downregulated inflammatory cytokines in Caco-2 cells, which reduced obesity-induced inflammation and promoted gastrointestinal health (Table 1) [15]. Additionally, they also show strong potential to inhibit the activity of *α*-glucosidase and pancreatic lipase [16]. In addition, quinoa polysaccharides, phytosterols, peptides, and phenolics exhibit a clear ability to prevent dyslipidemia, adiposity, and hyperglycemia. These bioactive compounds reduce lipid absorption and adipogenesis, increase glucose oxidation and energy expenditure, improve gut microbiota, and inhibit carbohydrate digestion. Quinoa offers outstanding attributes that support the dietary management of obesity [17]. In cultured colonic Caco-2 epithelial cells, quinoa polyphenols reduced interleukin (IL)−9, −1 and −8, and tumor necrosis factor (TNF) cytokines which inhibited obesity-induced inflammation and support gastrointestinal health in mice [18].

**Table 1:** Antiobesity potential of quinoa, chia, amaranth, nopal, avocado, pineapple and cacao

Crop	Bioactive compound	Antiobesity potential	Reference
Quinoa	Polyphenols	Reduce obesity-induced inflammation	[15]
		Inhibit the activity of *α*-glucosidase and pancreatic lipase	[16]
	Polysaccharides, phytosteroids, peptides	Reduce lipid absorption , adipogenesis, increase glucose oxidation, energy expenditure, improve gut microbiota, inhibit carbohydrate absorption	[17]
	Polyphenols	Inhibit obesity-induced inflammation	[18]
Chia	Rosmarinic acid	Downregulate adipogenesis	[9]
	*α*-linolenic acid	Improve insulin sensitivity and plasma lipid profile, reduce body fat	[20]
	Peptides	Reduce pro-inflammatory molecules (NF*κβ* and IL-6), PPAR*γ*, FAS, MAGL	[21]
	Cinnamic acids, flavonoids	Improve insulin resistance, liver damage	[23]
Amaranth	Soluble fiber	Regulate bowel movement, control hypercholesterolemia	[18]
	Leucin	Satiating effect, regulate insulin signaling pathway in muscle cells, and glucose homeostasis	[27]
	Protein, polyunsaturated fats (*α*-linolenic acid)	Weight loss, decrease liver cholesterol	[28]
Nopal (*Opuntia*)	Soluble fiber, polyphenols	Reduce fat absorption	[32]
	Dietary fiber	Improve gut microbiota	[33]
*O. robusta * fruits	Betanin	Reduce lipid peroxidation	[36]
Avocado	Phytochemicals	Induce satiety, regulate serum lipid profile, inhibit enzyme activity	[39]
	Ω -6 and -9 fatty acids	Reduce weight, BMI waist and hip circumferences, glucose, LDLc and HDLc levels	[40]
	Phenolic compounds	Induce satiety, reduce energy intake	[42]
	Mannhoheptulose	Improve insulin sensitivity	[43]
	Avocadene, avocadyne	Inhibit fatty acid oxidation, reverse pathologies associated to obesity	[44, 45]
	Oil	Reduce serum triglyceride, anti-inflammatory effect. Improve lipid metabolism, ameliorate inflammation oxidative status, reduce dyslipidemia	[41, 46]
Pineapple	Gallic and gentisic acid, epicatequin	Antiobesity and hepatoprotective properties	[50]
	Phenolic compounds	Restore fatty liver	[51]
	Bromelain	Regulate glucose metabolism and reduces insulin resistance	[53]
		Improve adipocyte metabolism	[55]
Cacao	Flavonoids	Ameliorate obesity and insulin resistance	[59]
		Induce satiety and reduce appetite	[60]
		Reduce lipid deposition and insulin resistance	[61]
	Proteins	Inhibit pancreatic lipase, reduced fecal triglycerides, lipids and fat absorption rate	[63]

*BMI* body mass index, *FAS* fatty acid synthase, *HDLc* high density lipoprotein cholesterol, *IL-6* interleukin-6, *LDLc* low density lipoprotein cholesterol, *MAGL* monoacylglycerol lipase, *MUFAs* monounsaturated fatty acids, *NFκβ* nuclear factor, *PPARγ* peroxisome proliferator-activated receptor gamma

### Chia

Chia (*Salvia hispanica*) has been known for over 5,500 years and was an important ingredient for the diets of Mayan and Aztec population. *S. hispanica* seeds are good source of *Ω*−3 and −6 polyunsaturated fatty acids as well as soluble dietary fiber (mucilage) (Fig. 1b), proteins and phytochemicals, mainly kaempferol. These bioactive compounds have antioxidant and satiety potential, as well as glucose and lipid lowering effect, among others, which may have a positive impact on the treatment of hypertension, obesity, some cardiovascular diseases, diabetes and cancer [4, 19]. Additionally, as gluten-free seed, chia can be considered for celiac diets [14].

In rats fed a sucrose-rich diet supplemented with chia, these seeds promoted reduction of body fat and modulated the intracellular lipid flow (fatty acid transport, oxidation and lipogenesis) in visceral adipose tissue. They also improved insulin sensitivity and plasma lipid profile, which are related to their *α*-linolenic acid (Ω−3 fatty acids) content [20]. (Table 1). Two chia peptides showed correlation with various amino acids of peroxisome proliferator-activated receptor gamma (PPAR*γ*), fatty acid synthase, and monoacylglycerol lipase. PPAR*γ* is linked to obesity and is associated to the negative regulation of nuclear factor-*κβ* (NF*κβ*) and pro-inflammatory molecules such as IL-6; also, they decreased the expression of both markers [21]. Caffeic and rosmarinic acid have been identified in chia leaf extracts and have potential to downregulate adipogenic factors [2, 22]. Also, these extracts are good source of cinnamic acids and flavonoids that provide anti-inflammatory and antioxidant properties thus improving insulin resistance and liver damage [23].

There are few studies over the effect of chia seeds on obesity; however, a classical study on this topic was published by Vuksan et al. [24]. Type 2 diabetic patients either with overweight and obesity followed a calorie-restricted diet for six months; one group diet was supplemented with chia (30 g/1000 kcal/day) and the control with oat bran (36 g/1000 kcal/day). Chia dietary intervention promoted more weight lose than control group; reductions in C-reactive protein and increase of adiponectin levels were observed. The diets were low in available carbohydrates and rich in antioxidants; moreover, further investigations are needed [6, 24].

### Amaranth

Amaranth (*Amaranthus* L.) has been rediscovered, and it is well-known that the native cultures of Mexico used this cultivar for their daily diets. Nowadays, it is cultivated in various countries of the world. Research studies have shown that it is one of the most valuable food crops with outstanding agronomic performance and nutritional value [25, 26]. In vitro and in vivo studies have demonstrated that the high content of leucin in this seed has a satiating effect, also regulates insulin signaling pathway in muscle cells, and glucose homeostasis, which are key characteristics to treat diet-induced obesity (Fig. 1c) [27]. Experimental studies using obese rats fed a diet containing nutraceutical foods like chia, curcumin and amaranth seeds promoted a significant loss of body weight and also LDL cholesterol, but no changes in serum leptin and insulin levels were observed (Table 1) [28]. Hence, amaranth may be considered as part of the strategies to prevent overweight and obesity.

In general, amaranth seeds and other pseudocereals may differ in size and shape to those from common crops, and as expected, they may exhibit different chemical and even physical properties. In amaranth and other pseudocereals the bran fraction is higher than that from the so-called traditional cereals; they also show higher levels of protein and fat. The kernels are also protected with a thin layer which may contain high amounts of protein [29].

The pericarp or seed coat, embryo and endosperm are the fundamental structures of most pseudocereals like amaranth, quinoa and kañiwa; oil, minerals and some proteins are mainly stored in the embryo whereas starch and most proteins are found in the endosperm. It is interesting to note that in the case of the color of amaranth´s pericarp it changes in a very remarkable way depending on the species; it can be dark brown, pale brown, black, white, cream, yellow, red or pink. The kernels of amaranth are smaller than cereal grains and those from quinoa [29, 30].

### ***Opuntia*** spp

**Fig. 2:**
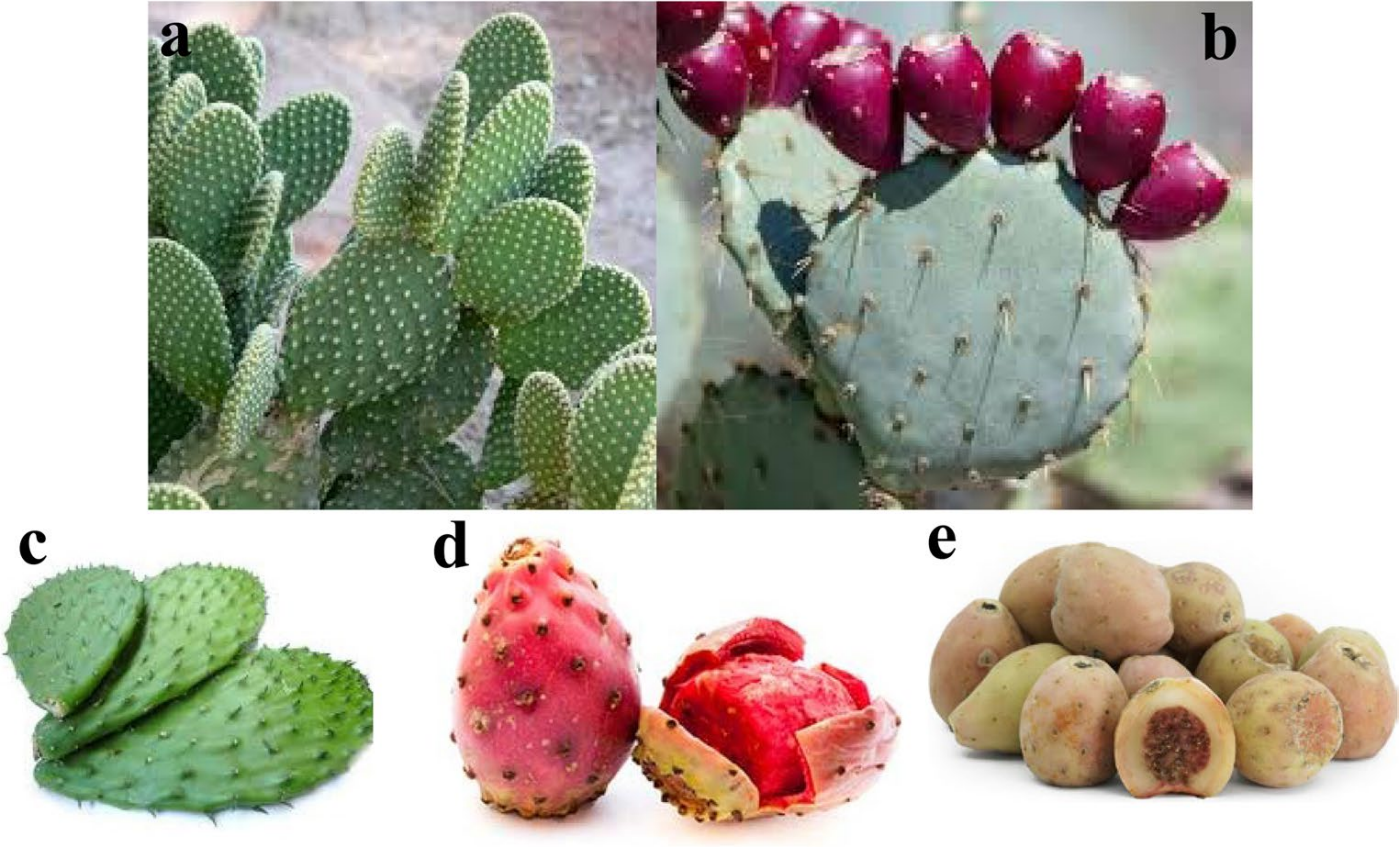
*Opuntia *spp*. *plant (**a**), *O. robusta *plant and fruits (**b**), “nopal” pads (**c**), prickly pear “tunas” (**d**) and *O. joconostle *fruit (**e**)

The great diversity of nopal plants has contributed extensively to the development of important cultures since pre-Hispanic times. Nopal (*Opuntia* spp.) (Fig. 2a, b) is the most used cactus for human and animal nutrition and food consumption mainly due to its physicochemical, nutritional and nutraceutical properties [8, 31, 32].

Nopal pad from *O. ficus indica* (Fig. 2c) is a rich source of total dietary fiber (57% dry weight basis) providing various benefits including gastrointestinal health. Caloric restriction, physical activity and a nopal-supplemented diet promoted changes in gut microbiota and improved the host metabolism in obese people [33]. In general, *Opuntia* cacti can improve lipid metabolism by inhibiting the absorption of fats in the gastrointestinal tract due to their soluble fiber and polyphenols levels, which contribute to a reduction of blood lipid levels (Table 1) [31]. In Mexico, *Opuntia* fruits are known as “tunas” (Fig. 2 d), they show a great diversity of colors with antioxidant potential. Red-purple fruits comprise betanin, a betacyanin that inhibits lipid peroxidation that acts as reducer of peroxyl radicals derived from unsaturated lipids of biological membranes [34, 35]. In *O. robusta* fruits, betanin showed the ability to scavenge nitrogen dioxide, radical responsible of low-density lipoproteins (LDL) oxidation [36];. In vitro and in vivo analysis showed that dietary *Opuntia* powders can regulate the adipocyte differentiation pathway and decrease fat absorption, in addition to changes in gut microbiota by developing certain bacteria and improving the host metabolism [33]. It has been demonstrated that the intake of *O. ficus indica* cladodes showed positive results in the management of obesity and the prevention of non-alcoholic fatty liver disease [36]. Other cactus species have also shown attractive nutraceutical potential such as *O. joconotle* fruits (Fig. 2e) [37]. The above mentioned functional capacities of *Opuntia* edible plants are key factors for health promoters in order to achieve antiobesity goals.

### Avocado

**Fig. 3:**
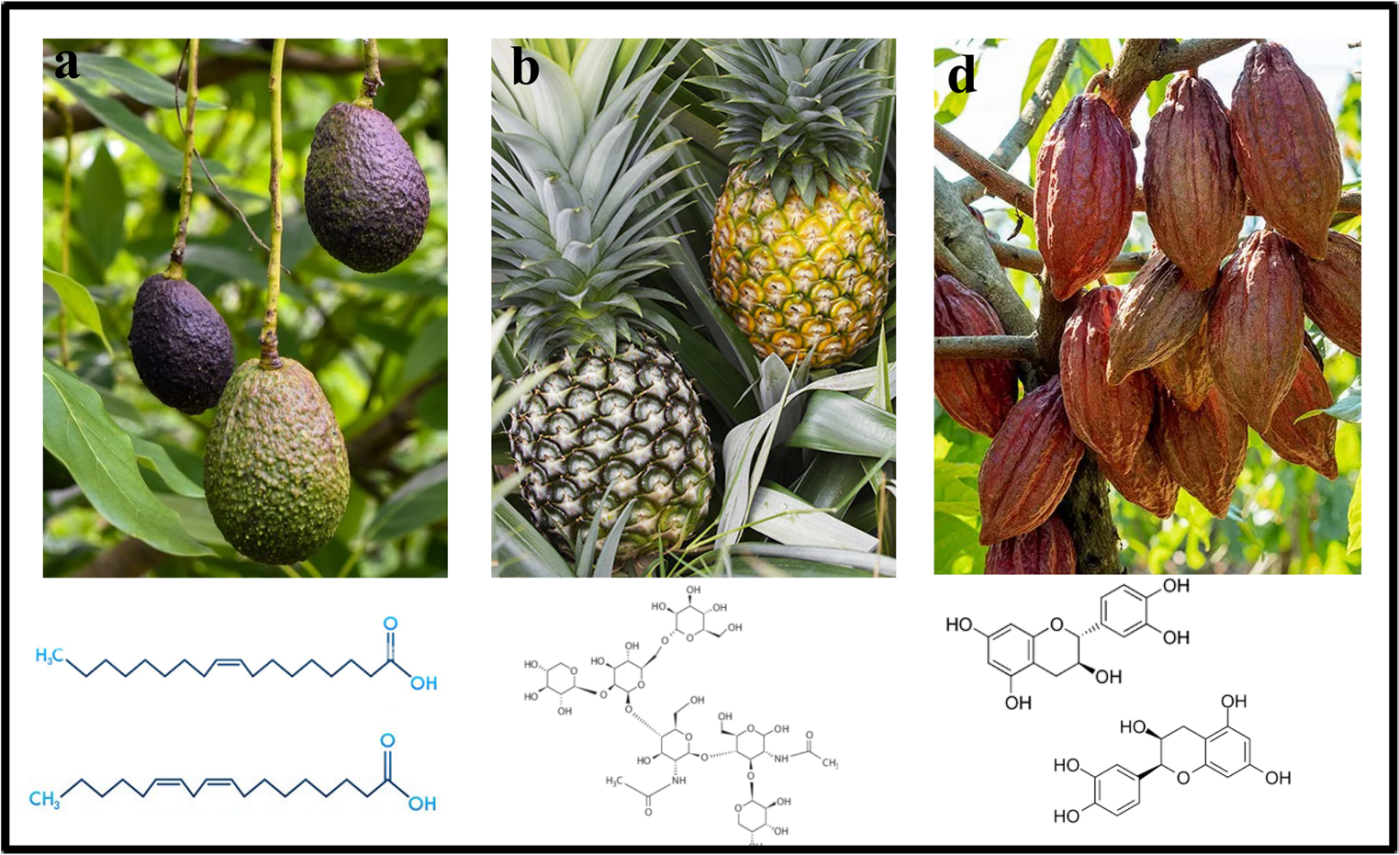
Avocado polyunsaturated fatty acids (**a**), pineapple bromelain (**b**), and cocoa polyphenols (**c**) with antiobesity capacity

Avocado (*Persea americana* Mill.) is a fruit native to the subtropical/tropical regions of Mexico and Central America. It is extensively produced and consumed around the world and there are more than 150 known species (Fig. 3a) [38]. Avocado is an excellent source of phytochemicals such as phenolic compounds, polyhydroxylated fatty alcohol derivates, phytosterols, carotenoids, tocopherols and alkaloids, which inhibit lipid peroxidation and have antiproliferative and antilipidemic capacity. These effects are related to their antioxidant potential, although they also regulate gene expression, protein activity and serum lipid profile, inhibit enzyme activity, cell cycle regulation, and very interestingly, they may induce satiety, between other functional characteristics [39].

*P. americana* oil is rich in bioactive substances like phytosterols, unsaturated fatty acids, antioxidant vitamins (carotenoids and tocopherol) as well as polyphenols. In individuals with hypercholesterolemia, the supplementation with avocado oil (10 ml/day) promoted positive changes in weight, BMI, waist and hip circumferences, glucose, LDL and high- density lipoprotein (HDL) cholesterol levels, attributed to its Ω−6 and −9 fatty acids [40]. This oil also improved insulin sensitivity, decreased hepatic fat accumulation and serum triglyceride levels, decreased oxidative markers and TNF-*α* and IL-1*β* in obese mice. The incorporation of avocado oil in the diet is an effective tool to alleviate the health consequences of obesity [41] (Table 1). Dietary supplementation with industrially generated avocado paste rich in phenolic compounds, stimulated satiety and reduced dietary intake in rat models. Increased plasmatic concentrations of glucagon-like peptide-1, adiponectin and leptin levels promoted satiety. Additionally, industrial avocado by-products are sources of phenolic compounds with in vivo satiety effect [42].

Unripe avocado naturally enriched in mannoheptulose (MH), a seven-carbon monosaccharide, mimics the positive effects of calorie restriction without the reduction of calorie intake. Obese patients subjected to a 12-week daily intake of unripe avocado extract rich in MH reduced insulin requirements in the oral glucose tolerance test [43]. Polyhydroxylated fatty alcohols from avocado, avocadene and avocadyne, inhibit fatty acid oxidation in pancreatic *β*-cells and skeletal muscle, thus reversing pathologies associated to obesity. They also modulate mitochondrial metabolism that induce leukemia cell death [44, 45]. Several avocado molecules are interesting for medical purposes. Avocado fatty acids are beneficial for the reduction of metabolic risk factors when are included in the daily diet. Therefore, their identification and purification could favor the advancement in pharmaceutical research to improve human health [46].

### Pineapple

Pineapple (*Ananas comosus* L. Merr.) is another native Latin-American fruit with outstanding functional properties and nowadays is widely cultivated in this region and in other countries, in view of its attractive taste in addition to the cited nutraceutical performance (Fig. 3b). Some important studies have demonstrated that pineapple comprise various nutraceuticals including dietary fiber, vitamins, and phenolic compounds, such as gallic and gentisic acid, and epicatequin [47–49]. They possess antiobesity, antioxidant, and hepatoprotective properties [50]. It has been found that the frequent consumption of pineapple fruit produces an improvement of the hepatic cholesterol metabolism which can restore fatty liver and protect vascular endothelium (Table 1) [51].

Pineapple has protective effect against vascular and hepatic abnormalities. The daily administration of pineapple reduced hepatic steatosis and improved vascular injuries in high cholesterol diet-fed rats. It also regulated blood and hepatic cholesterol metabolism in rats by diminishing cholesterol synthesis and downregulated SREBP2 and HMGCR expression due to the increased hepatic LDL-cholesterol uptake, and also upregulated low-density lipoprotein receptor expression [51]. Experimental studies have shown that gut microbiota may be modified due to the expression of some genes involved in obesity; and these changes are produced by the consumption of vinegar obtained from the fermentation of pineapple, thus, modulating inflammation and enhancing antioxidant effects in high-fat diet-induced obesity. Therefore, pineapple vinegar may be used as a potential alternative functional food for obesity treatment [52]. Bromelain, a complex mixture of proteolytic enzymes found in pineapple, regulates glucose metabolism and reduces insulin resistance, consequently it is a valuable factor for weight control and diabetes [53].

Research investigations have clearly shown that pineapple exhibits high levels of lipid-lowering and antioxidant properties. In vivo experimental studies have revealed that the daily consumption of pineapple is an excellent natural strategy for cardioprotection against hypercholesterolemia. Pineapple possesses antioxidant and lipid-lowering properties, and its daily consumption alleviates hypercholesterolemia-induced cardiac lipid peroxidation and pro-inflammation elevation [54]. Recent studies have demonstrated that a slight improvement of the hepatic cholesterol metabolism can restore fatty liver and the protection of vascular endothelium; and this outstanding effect is obtained by the frequent consumption of pineapple [55]. In brief, this behavior exerts a function in management of weight control. Also, pineapple shows high concentration of fatty acid esters from hydroxy fatty acids that have anti-inflammatory and insulin-sensitizing potential [56] providing metabolic benefits in patients with overweight and obesity.

### Cacao

Mayan population were the first in Latin America to cultivate the cacao (*Theobroma cacao*) plant (Fig. 3c). They prepared chocolate, a cocoa beverage made with hot water and cinnamon or pepper, which was called the “Food of the Gods” [57]. It is widely known that all Mayan groups used the whole cacao seeds as coins in the daily interchange of common goods. Cocoa contains around 300 components, including oleic, palmitic and stearic fatty acids, minerals, methylxanthines (theobromine and caffeine), tyramine, tryptophan, and serotonin, and polyphenols [flavanols (catechin and epicatechin)] [58]. Around the world, Latin America is the main source (80% approximately) of fine-flavor cacao, however no more than 5% of the global production is made with this type of cacao [59]. Greenberg and Buijsse [60] determined that smelling dark chocolate produced a satiety response and a reduction in appetite; hence, this simple action could help to prevent weight gain. Additionally, cocoa flavonoids produce metabolic effects inducing lipolysis, reduce lipogenesis, and increase adiponectin release reducing insulin resistance and lipid accumulation, hence alleviating obesity [61].

Dietary supplementation of theobromine (TB), a bitter alkaloid from cacao, induced browning of white adipose tissue and primary adipocytes in mice. Tanaka et al. [62] observed that TB supplemented in the daily diet promoted subcutaneous white adipose tissue browning, enhanced PPAR*γ*-induced UCP1 expression (in vitro) suggesting potential effects for the treatment of obesity. In rats fed a high-fat diet, cocoa protein exhibited antiobesity capacity by the inhibition of pancreatic lipase, reduced fecal triglycerides and lipids, and fat absorption rate [63] (Table 1). Yamashita et al. [64] found that a cocoa extract rich in methylxanthines decreased the accumulation of fat in adipocytes by downregulating the expression of adipocyte differentiation regulators within AMP-activated protein kinase activation, which is a central regulator of energy homeostasis, thus lowering the risk of obesity. Cacao polyphenols (CP) have the ability to suppress low-density lipoprotein oxidation and cholesterol uptake. Rats fed chocolate rich in CP showed significantly less accumulation of fat in the liver than the control group. Hence, CPs aid in the prevention of fatty liver disease highly associated to obesity and its related diseases [65].

## Conclusions

Nowadays, obesity is a major epidemy worldwide and the growing interest on its prevention and treatment using functional foods and nutraceuticals has increased the research on the effects of selected ancient Latin-American edible plant foods, such as those considered in this work.

These crops used in different ways for foods, medicines and industrial products, since old times, are receiving increasing scientific attention especially in the last few decades as sources of bioactive compounds in the management of overweight and obesity of human beings, and to reduce their side effects. Therefore, the incorporation of Latin-American crops in the daily diet may improve the quality of life at a global level.

Additionally, the economic costs of obesity, and even overweight itself and their comorbidities such as fatty liver disease, insulin resistance or diabetes, or cardiovascular effects are showing increasing trends; thus, it is becoming rather difficult for low-, medium-, and high-income societies to face these challenges at worldwide level.

It is now a good opportunity to emphasize the importance of the involvement of the key bio-components, extracted from selected Latin-American cultivars in multidisciplinary research challenges, novel and innovative, in biomedicine, nanotechnology, nutrigenomics, and other recent omics fields. Under these circumstances, outstanding and impressive results are expected to be produced in a short and medium time periods.

## Data Availability

No datasets were generated or analysed during the current study.
